# Information and Communication Technology for Managing Social Isolation and Loneliness Among People Living With Parkinson Disease: Qualitative Study of Barriers and Facilitators

**DOI:** 10.2196/48175

**Published:** 2024-01-17

**Authors:** Gomathi Thangavel, Mevludin Memedi, Karin Hedström

**Affiliations:** 1 Centre for Empirical Research on Information Systems Örebro University School of Business Örebro Sweden; 2 College of Business Alfaisal University Riyadh Saudi Arabia; 3 Department of Communication, Quality Management and Information Systems Mid Sweden University Sundsvall Sweden

**Keywords:** social isolation, loneliness, Parkinson disease, ICT, information and communication technology

## Abstract

**Background:**

Parkinson disease (PD) is a complex, noncurable, and progressive neurological disease affecting different areas of the human nervous system. PD is associated with both motor and nonmotor symptoms, which negatively affect patients’ quality of life and may cause changes in socialization such as intentional social withdrawal. This may further lead to social isolation and loneliness. The use of information and communication technology (ICT) plays an important role in managing social isolation and loneliness. Currently, there is a lack of research focusing on designing and developing ICT solutions that specifically address social isolation and loneliness among people living with PD.

**Objective:**

This study addresses this gap by investigating barriers and social needs in the context of social isolation, loneliness, and technology use among people living with PD. The insights gained can inform the development of effective ICT solutions, which can address social isolation and loneliness and improve the quality of life for people living with PD.

**Methods:**

A qualitative study with 2 phases of data collection were conducted. During the first phase, 9 health care professionals and 16 people living with PD were interviewed to understand how PD affects social life and technology use. During the second phase, 2 focus groups were conducted with 4 people living with PD in each group to gather insights into their needs and identify ways to manage social isolation and loneliness. Thematic analysis was used to analyze both data sets and identify key themes.

**Results:**

The results showed that the barriers experienced by people living with PD due to PD such as “fatigue,” “psychological conditions,” “social stigma,” and “medication side effects” affect their social life. People living with PD also experience difficulties using a keyboard and mouse, remembering passwords, and navigating complex applications due to their PD-related physical and cognitive limitations. To manage their social isolation and loneliness, people living with PD suggested having a simple and easy-to-use solution, allowing them to participate in a digital community based on their interests, communicate with others, and receive recommendations for social events.

**Conclusions:**

The new ICT solutions focusing on social isolation and loneliness among people living with PD should consider the barriers restricting user’s social activities and technology use. Given the wide range of needs and barriers experienced by people living with PD, it is more suitable to adopt user-centered design approaches that emphasize the active participation of end users in the design process. Importantly, any ICT solution designed for people living with PD should not encourage internet addiction, which will further contribute to the person’s withdrawal from society.

## Introduction

### Background

Parkinson disease (PD) is a complex, noncurable, and progressive neurological disease affecting different areas of the human nervous system. PD negatively impacts the ability of people to perform their daily activities. PD is most notably characterized by visible motor symptoms such as rigidity, tremor, slowness of movements, and impaired balance and coordination while walking [1-3]. PD is also associated with nonmotor symptoms such as sleep disturbances, problems with swallowing, communication issues, anxiety, and depression [4]. These motor and nonmotor symptoms cause changes in socialization such as intentional social withdrawal, that is, avoiding social participation and interactions with people [5,6]. These behavioral shifts may be prompted by various factors, including decreased facial expressions and slurred speech and may lead to social isolation and loneliness [7]. Both social isolation and loneliness contribute to reduced quality of life and well-being [8,9]. Social isolation refers to the objective lack of social connections, while loneliness is the subjective state characterized by negative feelings about having a lower level of social contact than desired [10]. Hence, understanding the complex interplay between motor and nonmotor symptoms and their impact on social interactions is crucial and may have a profound effect on quality of life of people living with PD.

PD predominantly affects individuals aged older than 50 years, and mean age of onset is around 60 years [1,11]. Approximately 1% of people aged older than 60 years and more than 3% of those aged older than 80 years are affected by this disease [1,12]. Currently, clinical examinations are done at infrequent time intervals during hospital clinical visits. Such examinations provide only a snapshot of PD symptoms at that point in time and do not completely reflect a patient’s daily living, including his or her social activities. Unfortunately, the medical community has a limited understanding of social isolation and loneliness and their impact into the health of their patients and lacks effective interventions to evaluate and integrate them into routine clinical practice [13].

The use of information and communication technology (ICT) plays an important role in managing social isolation and loneliness [14,15]. For example, videoconferencing platforms such as Zoom (Zoom Video Communication, Inc) and Skype (Skype Technologies [Microsoft]), allow individuals to connect with their friends, family, and colleagues in real time through video calls. Similarly, social media platforms such as Facebook (Meta Platforms, Inc) and X, previously named Twitter (X Corp), provide a space for people to connect with their loved ones remotely. It is, however, important to recognize that one-size-fits-all solutions may not be effective. Instead, solutions need to more tailored to the specific needs of different individuals and groups. The diverse range of specific needs, experiences, and preferences must also be met within these groups [16,17].

ICT solutions targeting people living with PD are mainly developed to monitor the disease, assess symptoms to provide optimal and individualized treatments [18-20], and empower the users to manage their disease [21]. A few more studies also exist on analyzing online discussion forums for people living with PD [22,23]. Further, few studies focused on improving the quality of life and well-being among people living with PD [24,25]. There needs to be more research focusing on designing and developing customized ICT solutions that specifically address social isolation and loneliness among people living with PD. Additionally, due to PD symptoms, some people have issues accessing online peer support or using technology [22]. So, it is important to understand the barriers faced by people living with PD regarding socialization and technology use while developing an ICT solution for managing social isolation and loneliness.

### Literature Review

There are different barriers associated with PD symptoms that may cause social isolation and loneliness. A scoping review study analyzed the existing evidence regarding social withdrawal in PD and found that physical, cognitive, and psychiatric symptoms of PD and perceived stigma have reduced social activities among people living with PD [7]. The effects of PD on people’s social interactions were explored through in-depth interviews conducted in Tehran by Soleimani et al [26], who found out that “progressive physical disability,” “mood disturbances,” “shrinking of social activities,” and “secluding oneself” caused social disconnections. Similarly, another study investigated the impact of living with PD among 19 participants and found that participants compromised their social activities due to embarrassment of their changed persona and speech problems [27]. This embarrassment experienced by people living with PD were reviewed by Maffoni et al [28]. They reviewed 14 papers and synthesized social stigma into 4 constructs: “stigma arising from symptoms,” “stigma linked to relational and communication problems,” “social stigma arising from sharing perceptions,” and “caregiver’s stigma.” The construct “stigma arising from symptoms” includes not only the embarrassment due to visible physical symptoms but also the progressive loss of functionality. Similarly, stigma linked to relational and communication problems includes oral communication issues (hoarseness and delayed thinking process) and body gesture issues (rigid and unexpressive face). Furthermore, Maffoni et al [28] classified the construct of “social stigma arising from sharing perceptions” into two distinct categories: (1) “perception exchange of others toward the patient” and (2) “perception exchange of patient toward the other.” The former one deals with the people living with PD considering other’s beliefs about their physical and mental status, whereas the latter one includes the perspective from people living with PD that they are a burden to their family due to a change or loss of social roles. Finally, the last construct includes the stigma experienced by family caregivers due to their ill family member’s embarrassing and visible symptoms [28].

Concerning ICT solutions that support social connection for people living with PD, an online support group was introduced during COVID-19 period through a series of weekly lectures by expert neurologists using Zoom [24]. The main goal of the support group was to improve the quality of life and prevent the worsening of PD symptoms by keeping the patients connected, educated, and empowered. Furthermore, Boulos et al [25] developed a web platform (LiveWell) to enable people living with PD to manage their health condition and promote well-being through social communities. In the platform, the main feature of social communities was designed as a discussion forum among people living with PD and facilitated interaction with carers and clinicians. A study in the United Kingdom analyzed different online discussion forums and communities for people living with PD and their caregivers [23]. They found that the discussion forums helped participants to share their knowledge and develop friendships. Further, a systematic review by Gerritzen et al [22] revealed that online discussion forums, either through the web or Facebook groups, keep people living with PD socially connected, create awareness of PD, and exchange social support among them. All the above studies, except Subramanian [24], did not focus on addressing social isolation and loneliness issues for people living with PD, despite supporting social connections. Due to this limitation, there was also a lack of knowledge about user needs for managing social isolation and loneliness.

Previous studies about technology solutions for social isolation and loneliness highlight the need for customized ICT solutions to suit the needs of individuals, specific groups, or degrees of social isolation and loneliness [16,17]. A review by Thangavel et al [17] analyzed customized ICT solutions targeting social isolation and loneliness among older adults. They found that there are different aspects contributing to reduced social isolation or loneliness among older adults using ICT solutions. Such aspects include social communication, social participation, a sense of belonging, companionship, and feelings of being seen. Increasing social communication through ICT helps older adults to enhance their social connections, while ICT that promotes social participation enables older adults to engage in social activities with others [17]. Further, increasing a sense of belonging, companionship, and feelings of being seen through ICT fulfils older adults’ emotional relationships and reduces their loneliness [17]. Given that PD is more commonly diagnosed aged older than 50 years, the findings from the review on customized ICT solutions targeting social isolation and loneliness among older adults may have implications for developing similar solutions for people living with PD.

### Objective

This study investigates the multifaceted barriers experienced by people living with PD in the context of social isolation, loneliness, and technology use. Furthermore, this study also investigates the social needs of people living with PD, which can facilitate the design and development of potential ICT solutions to manage social isolation and loneliness among people living with PD.

Therefore, the study aims to address the following research questions (RQs).

1. What are the barriers experienced by people living with PD in the context of social isolation and loneliness?
2. What are the barriers experienced by people living with PD in relation to their technology use?
3. What are the social needs of people living with PD to facilitate the design and development of ICT solutions for managing social isolation and loneliness?

The expected results of this study are twofold. First, a comprehensive understanding of the barriers and challenges faced by people living with PD in the context of social isolation, loneliness, and technology use will be obtained. This knowledge will inform the development of customized ICT solutions and strategies that effectively target these specific challenges. Second, the study will identify the social needs of people living with PD, enabling the formulation of an evidence-based framework for designing ICT solutions that cater to these needs. Through these outcomes, we aim to provide valuable insights that can lead to enhancing the well-being and social connections of people living with PD.

## Methods

### Study Scope and Framework

This qualitative study extensively examined the multifaceted barriers encountered by people living with PD in the context of social isolation, loneliness, and technology use. With people living with PD as the main focus, health care professionals’ perspectives were also considered to gain a holistic understanding of the disease’s impact. By encompassing these diverse participant categories, a thorough exploration of the disease’s impact was achieved. The research, carried out in Sweden, aimed to shed light on the social experiences of people living with PD. Furthermore, the study explored the social needs of people living with PD, which could serve as a foundation for potential ICT solutions aimed at alleviating social isolation and loneliness. The study framework depicted in Table 1 encompasses the phases of research, the categories of participants involved as sources of data, study objectives, research methods for data generation, and an approach to analyze the generated data, providing a comprehensive overview of the research process. In total, 2 distinct phases of data collection were undertaken to achieve the study objectives. In the initial phase, our focus centered around understanding the diverse barriers arising from PD diagnosis and its implications on social isolation and loneliness through interviews with people living with PD and health care professionals. Additionally, we explored the impact of PD on the technology use of people living with PD. Subsequently, the second phase involved facilitating focus groups with people living with PD to identify their social needs, providing insights crucial for the development of strategies to alleviate social isolation and loneliness.

**Table 1:** Study framework.

Phases of research and participant category	Period	Objectives	Research method	Data analysis approach
Phase 1: health care professionals	November 2019	To understand how PD^a^ affects a person’s social life and quality of life	Interview	Thematic analysis
Phase 1: people living with PD and relatives	April to June 2021	To understand the barriers of being diagnosed with PD and how that relates to social isolation and loneliness. To understand how PD affects the technology use of people living with PD	Interview	Thematic analysis
Phase 2: people living with PD	January 2022	To understand the social needs of people living with PD to facilitate the design and develop ICT^b^ solutions for managing social isolation and loneliness	Focus group discussions	Thematic analysis

^a^PD: Parkinson disease.

^b^ICT: information and communication technology.

### Study Participants and Recruitment Procedure

The health care professionals who took part in the study included physicians, nurses, and rehabilitation team (physiotherapists, occupational therapists, and social workers). These professionals were actively associated with the neurology department at a university hospital in Sweden, where they provided dedicated care and support to people living with PD. To ensure a comprehensive and insightful participation of health care professionals, a neurologist who was part of a previous study played a pivotal role. The neurologist assisted us in sending invitations to coworkers within the neurology department. Furthermore, the neurologist shared contact information of interested health care professionals and facilitated the scheduling of interviews based on each expert’s availability.

We extended invitations to people living with PD who met the specific inclusion criteria, which included being aged older than 50 years, having been diagnosed with PD, and demonstrating the ability to understand and express willingness to sign the informed consent form. People living with PD with severe cognitive problems, which could potentially impact their ability to provide reliable responses, were excluded from participation. Further, people living with PD were also invited to bring relatives or another close person if needed. The information from those relatives was used to complement the information given by the people living with PD. Invitations to participate in the study were facilitated through the national Parkinson association, which played a crucial role in identifying and reaching out to eligible individuals who fulfilled the study’s inclusion and exclusion criteria. The association’s support was instrumental in connecting us with people living with PD from 2 different regions in Sweden.

### Data Collection

#### Interviews With Health Care Professionals

Semistructured interviews were conducted with open-ended questions related to PD focusing on the quality of life and social aspects. In addition, general questions related to treatments or therapies and ICT used in relation to PD were included (see the interview guide in Multimedia Appendix 1). The interviews with physicians and nurses were conducted individually, whereas the interview with the rehabilitation team were a group interview, due to their work nature as a team. In total, the interviews were conducted with 2 physicians, 2 nurses, 2 occupational therapists, 2 physiotherapists, and 1 social worker. The interviews took place at the university hospital and were recorded with their consent; on average, each interview lasted approximately 45 (SD 8.62) minutes.

#### Interviews With People Living With PD

Individual interviews with people living with PD were conducted during the period from April to June 2021. Due to the COVID-19 pandemic, physical meetings were not possible, so online interviews were conducted via Zoom. For the participants who did not have previous experience with Zoom, we also sent a link with information about how to join Zoom meetings. We also gave telephone numbers and meeting IDs to connect to the Zoom meetings. Further, 2 participants connected through this feature. Questions were asked about their background, challenges of living with PD, social isolation and loneliness, technology use, and issues. Questions related to social isolation and loneliness were adapted from Cornwell and Waite [29] since their study included both isolation and loneliness aspects (see the interview guide in Multimedia Appendix 1). As the interview took place during the COVID-19 pandemic, the discussion did not center around their experiences of socializing amid the pandemic. Instead, the people living with PD were requested to share their perspectives on how PD had impacted their socialization. Interviews were conducted in the language (English or Swedish) based on the preference and comfort of the participants. In total, 18 interviews were conducted, each taking 35 (SD 6.06) minutes on average. All the interviews were recorded with the participants’ consents. However, it’s important to note that 1 participant chose to withdraw from the study after their interview.

#### Focus Group With People Living With PD

As a follow-up, 2 focus group discussions were conducted with people living with PD in January 2022 to understand how they would manage or would like to manage social isolation or loneliness with the use of ICT. Invitations were sent to all the people living with PD who participated in the initial interviews. Based on their acceptance and availability, the focus groups were arranged. In total, 4 individuals participated in each group using Zoom. Further, 1 group preferred the Swedish language, while the other chose English. Each group discussion was recorded with their consent and lasted about 1 hour and 30 minutes. Discussions were concentrated around 3 topics: coping mechanisms for social isolation and loneliness; social communication, social participation, and sense of belonging; and technology barriers and needs. These topics were selected based on the insights from the results obtained during initial individual interviews with people living with PD and findings from a previous literature review [17].

### Data Analysis

The audio-recorded data were transcribed, and later, the Swedish interviews were translated into English because the main author needed help in understanding Swedish. The interview data was analyzed using an Excel (Microsoft) sheet following the thematic analysis process [30]. Initially, transcripts were manually coded line by line based on the content by the first author. For example, codes such as challenges, diagnosis, tiredness, anxiety, apathy, embarrassment, stigma, social connection, social network, events information, and awareness were formed. The codes were then sorted and collated based on the RQs, that is, barriers to socialization, barriers to technology use, and needs for managing social isolation and loneliness. Subsequently, the sorted codes under each RQ were further categorized into themes by inductive and deductive approaches as shown below by the first author.

The codes linked to the RQ1 and RQ2 were further categorized by organizing the codes together based on the relationship between codes. For example, apathy and anxiety were put together since both are related to the psychological conditions of a person. The part related to stigma is further categorized using the social stigma model from Maffoni et al [28].

For the codes related to RQ3, we classified using the aspects identified by Thangavel et al [17], which include social communication, social participation, and a sense of belonging. For example, events information is categorized under the social participation theme since information about events enhances social activities with others.

Finally, all the authors reviewed and refined the themes until a consensus was reached. For example, under RQ2, the code “learning difficulties” was originally mapped under the theme “ageing concern.” However, through our collaborative review, we collectively agreed that this code would be more appropriately categorized under “cognitive limitations.” Further, we also extracted the quotes that capture each theme’s essence.

### Ethical Considerations

This study followed the Swedish ethical guidelines and received approval from the Swedish Ethical Review Authority (2020-05855). For all the participants, the study was fully explained orally and in written form, and their written or digital informed consent was obtained before the data collection. Participation in the study was entirely voluntary and no compensation was provided for their involvement. Further, the participants had the right to withdraw from the study at any time, without giving any reasons.

## Results

### Participants’ Characteristics

Information about the people living with PD and health care professionals who participated in this study is described in Tables 2 and 3, respectively. The average age of people living with PD in this study is 67 (SD 5.88) years. Out of 17 interviews, 16 were people living with PD and 1 was a relative. This 1 relative represented her recently deceased mother, diagnosed with PD for 17 years. Apart from that, 2 other relatives (spouse) joined along with people living with PD. At the time of the interview with people living with PD, the participants had been diagnosed with PD for an average of 8 (SD 4.52) years. Further, 4 people living with PD were living alone and others were with their spouses.

**Table 2:** Characteristics of people living with PD^a^ (n=16).

Characteristics		Value, n (%)
**Age group (y)**		
	56-60	3 (19)
	61-65	5 (31)
	66-70	3 (31)
	>70	5 (19)
**Gender**		
	Women	7 (44)
	Men	9 (56)
**Living situation**		
	Alone	4 (25)
	With partner	12 (75)
**Working status**		
	Retired	13 (81)
	Working	3 (19)
**Years since diagnosis**		
	≤5	6 (38)
	6-10	4 (25)
	11-15	6 (38)

^a^PD: Parkinson disease.

**Table 3:** Characteristics of the health care professionals (n=9).

Health care professionals	Value, n (%)
Physicians	2 (22)
Nurses	2 (22)
Rehabilitation team	5 (56)

### Emerged Themes

The outcomes of the interviews and focus group discussions yielded distinct themes in response to each RQ. For RQ1, the identified themes encompassed “fatigue,” “psychological conditions,” “social stigma,” and “medication side effects.” Notably, the subthemes within the “social stigma” category were developed following the social stigma model proposed by Maffoni et al [28]. Furthermore, addressing RQ2, the exploration of barriers associated with the use of ICT revealed themes centered around “physical limitations” and “cognitive limitations.” Lastly, RQ3 centered around the user needs for managing social isolation and loneliness, were categorized into “increase social connections,” “increase social participation,” “increase a sense of belonging,” and “other suggestions.” Some of the example codes associated with each theme are described in Table 4.

**Table 4:** Themes, subthemes, and codes.

RQ^a^, themes, and subthemes		Example codes
**1. Barriers in the context of social isolation and loneliness**		
	Fatigue	Energy depletion, slowed movements, and extreme tiredness
	Psychological conditions	Anxiety (fear about future and excessive worry) or apathy (lack of initiative)
	Social stigma Arising from symptoms Due to relational and communicational problems Arising from sharing perceptions Perception of others toward people with PD^b^ Perception of people with PD toward others	Embarrassment, shame, and stigma
	Medication side effects	Gambling, hallucinations, and impulse control disorder
**2. Barriers in relation to use of ICT^c^**		
	Physical limitations	Difficulties using keyboard and mouse
	Cognitive limitations	Not remembering passwords, complex to navigate, and learning difficulties
**3. User needs for managing social isolation and loneliness**		
	Increase social connections	Connect similar interest people, video chat, and group establishment
	Increase social participations	Events information, create event, and recommendations
	Increase a sense of belonging	Awareness, community forum, information from experts, and tips and suggestions
	Other suggestions	Simple, easy-to-use, and technology guidance

^a^RQ: research question.

^b^PD: Parkinson disease.

^c^ICT: information and communication technology.

### Barriers in the Context of Social Isolation and Loneliness

#### Overview

Results from the interviews with people living with PD show that most people living with PD withdraw from socializing with others due to PD and its symptoms. In total, 13 people living with PD reported being socially isolated; 10 people living with PD also expressed feeling lonely at some point of time due to PD. Further, 3 out of 4 people living with PD who live alone were feeling lonely. Apart from this, the following factors which cause social withdrawal were identified.

#### Fatigue

One of the main causes identified by health care professionals and people living with PD is extreme tiredness due to PD. Therapists pointed out that this was attributed to the motor fluctuations experienced by the patients on that particular day.

If they are tired due to physical and cognitive fluctuations, it will obstruct their routine and plans...one day they feel really good, and the next day they decide to eat food at a restaurant but motor fluctuations on that day will make them tired and stop them from going out.

Physiotherapist 1

Most people living with PD also expressed feeling exhausted due to their deteriorating physical and mental abilities. Due to this experience, they avoided spontaneous meetings with others. Additionally, they expressed that they need to plan and organize everything based on their bodily needs.

I’m not able to decide myself when I want to meet people, Parkinson is deciding how I am able to go to a social meeting. The disease determines when and how and what I should be able to do. I am being imprisoned by the disease more and more.

Person living with PD 11

#### Psychological Conditions

People living with PD also experience an on-off state, that is, a switch between mobility and immobility occurs at the end of the dose of medication. During the off state, motor functions will deteriorate and concurrently, the mental well-being of people living with PD is significantly affected, leading to increased anxiety. The challenges presented by this fluctuation contribute to the development of psychological conditions, notably apathy (lack of initiative) and anxiety. For some people living with PD, these symptoms can result in a loss of interest in activities and a reluctance to engage in social interactions and outings.

...can also have mental on and mental off which is ability to think and no ability to think or being really anxious or not anxious. So, this can fluctuate with levels of medication. This can sufficiently repair their ability to engage in social activities.

Physician 1

...Yes, she isolated. She had friends, but she didn’t call them. She wouldn’t pick up the phone and I took friends to her house for coffee and then she enjoyed it and then it worked, but she would not take the initiative herself.

Relative 1

Due to the nature of this disease, they worry a lot about their balance while they walk. They are not going out thinking they cannot come back without other’s help.

Some patients get physically isolated due to anxiety they get that they cannot go back home on their own.

Physician 2

Further, this anxious nature also prevents people living with PD from meeting others diagnosed with PD. They worry that when they see others who have worse symptoms, they will have those symptoms later.

I get more depressed from going to the other meetings where you see those that are significantly worse. And actually, maybe it’s the same for them. They can imagine: “Why have I got this, and am not as healthy as the other?” So, it’s kind of not good for anyone.

Person living with PD 5

She didn’t want to see how sick they were, she was afraid because some of them...of course some were better than her, but some were terribly ill and that frightened her.

Relative 1

#### Social Stigma

##### Social Stigma Arising From Symptoms

Some of the most common visible symptoms of PD are tremors and drooling. A tremor (shaking) is an involuntary movement affecting part of their body, particularly the hands. Due to these visible motor symptoms, people living with PD expressed that they are embarrassed to show their symptoms in front of others.

I am very fond of going to the theatre, the opera, concerts, but sometimes I feel, no I can’t sit among people when my arms start shaking, so I am afraid that everybody can see. And it is difficult to concentrate on the music or whatever.

Person living with PD 14

This symptom makes people living with PD stay at home and avoid going out. Health care professionals also confirmed that this complaint is the biggest one they receive from their patients when enquiring about their social activities.

There are like emotions such as shame that can stop people from engaging. If you shake and you feel shame of that in a social situation, you may stop being part of the family dinner. If you start drooling because you don’t think of swallowing your saliva, you may avoid people, so people cannot see drooling and people think of you like the person you were in your better days. So, people do some social hiding there too.

Physician 1

Apart from the visible symptoms, some people living with PD felt uncomfortable because of their progressive loss of functionality and autonomy. Further, they felt bad about themselves and were not able to ask for help easily if they cannot do certain things like cutting meat or putting on shoes.

...I went by myself to a restaurant, and then I get the main course in and it’s a piece of meat, and I can’t cut the meat. And then I felt like this: “Yes, it was not so easy to ask for help.” Yes, I’ve thought it is. I've always said this: “Yes, but if I don't know anything, then I'll ask someone for help.” But it wasn’t that easy.

Person living with PD 6

##### Social Stigma and Relational and Communication Problems

Further, during the PD off state, some people living with PD mentioned that their thinking speed had decreased, making it challenging to find the right words to communicate with others.

That they don’t listen. They don’t hear what I say and that they don’t listen as much as before, on what I have to say. That’s the negative. And for my wife it was much worse, because people didn’t hear what she said and then when she said something, they either ignored it or they had to ask what she said, or the other was silent just to try to figure out what she had said, or someone could hear what she said. So, it was...that meant that she was more and more silent in groups, yeah, at the end there.

Person living with PD 10

The nurses and the therapist also mentioned that some patients have swallowing issues and difficulty eating due to PD, making them not go to restaurants with their family or friends.

Some patients have problems with talking, they have soft or hoarse voice, and very hard time to speak. And some patients have hard to swallow and that’s the reason they won’t go out to restaurants or family dinner or eat with other people.

Nurse 2

##### Social Stigma Arising From Sharing Perceptions

###### Overview

This category was also one of the major concerns raised by people living with PD. There were two different perceptions shared: (1) perception of others toward people living with PD, and (2) perceptions of people living with PD toward others (caregivers and family).

###### Perception of Others Toward People Living With PD

The people living with PD expressed that, opinions from their family or friends such as “it’s a terrible disease” or “you don’t fit in” make them upset and unwilling to socialize with their loved ones. Due to this perception, some people did not feel comfortable sharing information about PD with their friends.

...They don’t invite...they didn’t invite us, and don’t invite me for some activities where they think we don’t fit in because of the Parkinson. For example, some party with dance and so on, so I think naturally they choose which ones fit in to a party, and then they think we were less attractive.

Person living with PD 10

I think it’s a little bit embarrassing to tell new friends about this. My old friends, they know all about me, it’s not embarrassing at all, but if I meet a new...friend and then...they don’t know what Parkinson is and they don’t really understand.

Person living with PD 11

Further, in certain cases, their family and friends are not able to understand their illness and they avoid asking the people living with PD about their health condition.

...most of the time I put in tablets and stuff like that to be able to meet them, but they can’t, or don't really understand my illness and this. And they don't want to talk about it, don't want to know. My son he says only briefly: ‘How are you? Good, or bad’, like. And they don't want to know any more, and it's a little frustrating sometimes.

Person living with PD 4

###### Perception of People Living With PD Toward Others

Some people living with PD feel they have lost their personal identity. They want to be treated normally, just like they were treated before their diagnosis. Their self-esteem was shattered by others in the family. Their sense of belonging was lost in some cases. Further, people living with PD feel they burden their family since they depend on others for everything.

...It affects their psyche too, that I'm not feeling well. I lie down on the floor, they pick the yoga mat, they pick the ball. I would like to...have a separate room, but it is not possible, to isolate me when I exercise, and that they do not see me suffer.

Person living with PD 8

No matter what you suffer from, it is well that the surroundings have difficulty relating to how to treat the person who has received something, Parkinson’s or something else. But I want to keep my identity and not become a Parkinson patient...I want everybody to treat me as a person not as person with Parkinson.

Person living with PD 17

##### Medication Side Effects

Health care professionals highlighted that, at an advanced stage, due to more dosages of medications, there are side effects like hallucinations, behavioral problems, gambling addiction, and sex addiction.

...they have confusions or hallucinations due to the side effects of medicines. We get complaints from their partners. Because patients get suspicious if their partners go out and it affects their relationship.

Nurse 1

Due to these side effects, health care professionals further emphasized that patients may have problems with their life partners and their family members. Further, they expressed that some patients may be more prone to gambling or online shopping due to high dopamine effects. This impulsive control disorder and gambling makes the person withdraw from their social circle.

Due to increase in dopamine, they have side effects. They want to do some things repeatedly or totally focusing on something or become hyper – if they get some rewards. Some may lose money in gambling; some may do more shopping online.

Physician 2

#### Barriers in Relation to the Use of ICT

##### Overview

In addition to the difficulties faced by people living with PD in socializing, there are also obstacles related to their PD symptoms and aging that impede their ability to use ICT. These barriers are discussed below.

##### Physical Limitations

Due to Parkinson tremors and shaking symptoms, some people living with PD expressed difficulties in using a keyboard and mouse. With the shaking and tremor symptoms, keys were pressed wrongly, and sometimes the same key was pressed for a long time.

And then not only that, but you are fiddling with all the other letters as well, even if you don't want to at the same time, I think. That's a problem, I think too.

Person living with PD 6

Due to this issue, they needed help to complete the task. In the case of mouse handling, it was more difficult since the precision of the mouse pointer is important when they want to click something on the screen.

I don’t shake so much now, but I...sometimes when I shake, when I’m in an off- or on-state, to run the mouse is like whisking your cream to morning coffee...To move the mouse exactly when it’s a millimetre is hell sometimes.

Person living with PD 16

Furthermore, many people living with PD are unaware of accessibility options for keyboards such as “filter keys,” and “slow keys” to ignore repeated keys and slow down. Further, most of them do not know about alternative options like voice control. Some people living with PD expressed that it was difficult for them to change or use those options, even if they were aware.

I think it's possible that you can set on the keyboard how hard you have to press to make something happen. But it's not something I can do, and it's something like that that would make it easier, so that... it should be touch controls, of course, but it shouldn't be that it's enough to breathe on them to trigger a response.

Person living with PD 3

You mention voice control now, I haven't used it at all, I don't know there's anything like that. So that it is a small obstacle.

Person living with PD 15

##### Cognitive Limitations

Some people living with PD expressed that they could not remember codes or passwords because of cognitive decline symptoms. This situation also makes them anxious in some situations. Due to that, some people living with PD also needed help when the application was too complex to navigate. They explicitly stated that they were looking for help.

...passwords get very complicated. So, you need some support to make it simple and also to have someone to help out...I am getting a little bit nervous, and I fear that something will happen, that I would lose something, some information or...lock the data...the computer and so on.

Person living with PD 15

Whatever solution you have digitally...if it is complex...You need help sometimes. You need someone to ask.

Person living with PD 17

Some people living with PD also expressed that learning a new technology is difficult due to aging. They also conveyed that recent technology developments are not user centered and did not consider their requirements while designing new technologies.

...it’s more difficult to learn about new technology today, if I compare for...when I was younger. But I think that’s...maybe...I don’t think it’s not just about Parkinson as much as the age.

Person living with PD 7

...those who make these computers and technologies, they are not...the users. They should ask us who use these devices, ask us what we need and how and so on, but they are not interested in other than their own investors.

Person living with PD 14

#### User Needs for Managing Social Isolation and Loneliness

##### Overview

In order to manage their social isolation and loneliness, people living with PD offered several suggestions that could guide the development of relevant ICT solutions.

##### Increase Social Connections

The people living with PD wanted a simple and easy-to-use digital solution that could help them connect with other people with similar interests or hobbies and establish themselves as a group.

...so it’s easy to pick up and see where you can find people that have the same interest as you have, for example golf, tennis, or cars or whatever. That could be...easy way to find new friends...If you can find other people that way, as a...maybe you can...easier to take new contact with new people and...learn from them as well...and share experience.

Person living with PD 7

In that way, they can communicate about their interests, share their understanding of what they know, and learn from others. In the long run, this group establishment can also help people living with PD who are lonely to rely on and interact with someone in the group whenever they want to have an emotional connection.

It’s this that you have someone you can talk to when needed, huh, that when you feel lonely you would have someone to call. Oh, I do, so I don't really miss it, but maybe that's what's most important, that you have someone to talk to.

Person living with PD 2

Further, most of them recommended that digital solutions should facilitate connections not only among people living with PD but also with individuals who share similar interests.

...someone has to connect people in one way or another, because if you sit on your own and feel lonely and have Parkinson, you are not that likely to establish some kind of contact with other people. And I mean, that might be a good idea to connect to people with Parkinson, but it could also be a good idea to connect with people that don’t have Parkinson.

Person living with PD 3

Since many of them become exhausted easily because of PD, they expressed that having a digital solution to communicate with groups through video chats would help them interact with others without losing much energy.

it’s easier to meet someone by the computer, it doesn’t require so much energy. You don’t have to go somewhere and take the bus or walk. You can sit at home, and you can meet people, talk to them.

Person living with PD 11

##### Increase Social Participation

Apart from initiating group communication, some also suggested having recommendations about events (both digital and offline) and clubs and deals based on their locality and their interests or hobbies. The people living with PD also suggested a solution that helps them to create an event if they would like to give training or share their expertise with others. Currently some people living with PD were unaware of the existing classes such as ballet, boxing, and dance for PD.

Training...if you find training, if you can for example show us where...for example, some people don't know. Still some Parkinson's patients do not know that there is dance, ballet dance for Parkinson's. If you refer to offers, that one pays, free etc...It also sounds perfect. Very good.

Person living with PD 8

They suggested that information should include the time, date, location, and whether it is a recurring event. This information helps them to plan in advance. It makes them participate in social activities. Further, they stated that this kind of information would help people living with PD who have difficulties taking the initiatives to go out.

...this communication about different activities, what times...time it starts, where is it, how many times, how many weeks. So, if you want to be in touch with other people, then I think it’s the best to know when and where.

Person living with PD 14

##### Increase a Sense of Belonging

In order to create a sense of belonging and feeling valued, many people living with PD suggested having a community forum to share information about their experience with PD. In that way they feel they can help others and have a sense of purpose. They are also looking for the reliable latest information about PD from experts.

When I was diagnosed three years ago, I told my children and then, it was not more than week or fortnight until they had googled and were very well informed and told me about it. So, information to people is the best way to feel belonging.

Person living with PD 13

..there are several people who call me who are Parkinson sick, and are worried, and have not received the care that they want...you have to help, give them tips and suggestions...create a forum where you can ask questions, and also answer others...it could be...it’s probably quite rewarding for a lot of people, I think, who are alone and don’t have anyone to talk to.

Person living with PD 2

##### Other Suggestions

The people living with PD also suggested having a simple, easy-to-use solutions where they do not need to remember any passwords or codes. Some people living with PD felt that proper technology help and guidance would help them better use computer, phone, and tablets.

But what I really want to stress is that it should be simple to use, and I think also you need to have support if something happens...I always say that crucial aspect is that you have to...to have user-friendly technology and both physically, using the fingers, and then also mentally, that you should have systems where you do not have difficult password, that makes you nervous and so on.

Person living with PD 15

## Discussion

### Principal Findings

The results of this study describe difficulties faced by people living with PD in the context of social isolation and loneliness along with their technology use. In addition, the results highlight the high variability among people living with PD for intended social withdrawal. For some people living with PD, it was the fatigue caused by PD that restricted their participation, whereas the psychological conditions and social stigma due to PD played a role for others. The social comparison theory [31] may explain the anxious nature observed in certain people living with PD, as they worry about encountering individuals with more advanced symptoms. Alternatively, seeking interactions with peers facing similar experiences may provide comfort and support, fostering a sense of belonging and reducing isolation. Understanding these influences can inform targeted interventions, such as support groups, to address the social and emotional needs of people living with PD effectively. When compared with social stigma constructs such as “stigma arising from symptoms,” “stigma linked to relational and communication problems,” “social stigma arising from sharing perceptions,” and “caregiver’s stigma” [28], we found that the people living with PD experienced stigma due to their symptoms, relational and communication problems, and also due to sharing perceptions with others. However, the relatives who participated in this study did not express any stigma (“caregivers’ stigma”) because of their family member’s condition. This situation may be because very few relatives participated in this study and no question was asked about their life experiences separately. We also found that the medication side effects were related to increased impulse control disorder (eg, gambling), which could further lead to social withdrawal which was not highlighted in previous studies [7,26,27].

Not surprisingly, we found that the majority of the people living with PD in our study stated that they experienced difficulties using technology, especially, the keyboard and the mouse due to their motor symptoms. This aligns with previous research that has shown people living with PD encountering problems with typing and using a mouse [32,33]. Adopting alternate input devices or adapted (larger keys) keyboards and mouse can be instrumental in addressing these challenges and making ICT solutions more accessible and user-friendly for people living with PD. Further, we also found out that due to PD, people living with PD also have cognitive decline making it difficult to remember passwords and complete complex tasks. Furthermore, there is a lack of IT support to help people living with PD in case of any issues. Some people living with PD explicitly stated that they did not know where to find IT support. Besides PD symptoms, aging also contributed to difficulties in using a computer, such as learning new technologies.

As mentioned earlier, most of the ICT solutions such as online support groups or discussion forums are not tailored to the needs of people living with PD for managing social isolation or loneliness [22,23]. This point was also raised as a concern by people living with PD, who mentioned that including them during the design phase of new solutions is essential. Therefore, given the wide range of needs and barriers experienced by people living with PD, it is more suitable to adopt user-centered design approaches that emphasizes the active participation of end users in the design process [34,35].

Our results demonstrated that the solutions should consider the barriers faced by people living with PD which restrict their social activities. For example, some may experience “apathy” and withdraw from social activities, hence the ICT solution should address this and help them to socialize by incorporating features that actively encourage socialization and engagement. This can be done by providing features such as customized content, online community, online events, and reminders or prompts for people living with PD to participate in social activities. Further, to enhance the accessibility of ICT solutions for people living with PD, developers should consider incorporating features like voice recognition, enabling users to convert spoken words into text or commands. This feature could be particularly useful for people living with PD who have problems typing or using a mouse, allowing them to dictate text and navigate the solution using voice commands. However, it is important to acknowledge that speech-related challenges are prevalent among some people living with PD, and standard voice recognition technologies may not always be effective in recognizing weak or impaired speech. Therefore, specialized software and adaptations should be developed to cater to the unique speech needs of people living with PD, ensuring that the ICT solutions are truly inclusive and beneficial for all users. Further, our people living with PD emphasized the need for simple easy-to-use ICT solutions, which has also been highlighted in many studies [20,36].

Concerning the needs highlighted by people living with PD, they emphasized that ICT solutions should not only be used by people living with PD but should include everyone. Currently, discussion forums or platforms designed for people living with PD mostly include people living with PD alone or may also include carers and health care professionals [23,25]. Furthermore, some people living with PD wanted information about public events near their residence to be customized based on their interests. In contrast, some people prefer communicating online with others based on their interests. For people who experience social stigma, connecting with others who have similar experiences with the disease can help the affected individuals to overcome their stigma [37]. Hence the PD people’s suggestion of an online discussion forum not only helps to create a sense of belonging but may also help them to overcome their stigma by coming to know other people’s experiences. These findings indicate that ICT solutions need to be adapted and tailored based on the barriers experienced by people living with PD and their ICT needs, in order to facilitate managing their social isolation and loneliness.

The study also revealed a significant finding related to impulse control disorder caused by medication side effects highlights the importance of designing ICT solutions that do not promote internet addiction. Excessive internet use can lead to social withdrawal and further isolate individuals from society [38,39]. Therefore, it is crucial for technology developers and designers to consider the potential impact of their solutions on users’ behavior and well-being. Incorporating features that prioritize meaningful engagements instead of addictive features such as aimless scrolling, automatic video playback, and gamification elements (such as points and rewards) can promote positive social connections and reduce the risk of internet addiction.

### Strengths and Limitations

The main strength of this study was the inclusion of both people living with PD and health care professionals. This approach has provided unique insights and perspectives that might not be accessible to researchers who do not have direct experience with the disease. Furthermore, this inclusion allowed us to better understand the barriers faced daily by people living with PD. For example, the people living with PD shared their personal experiences and explained how PD had affected their social life, while the health care professionals provided medical explanations for these challenges. Further, the health care professionals’ involvement helped us to identify a new barrier “medication side effects.” Overall, involving both people living with PD and health care professionals in this study gave us a complete picture of the challenges that people living with PD face and helped us to identify important issues that might have been missed if we had only spoken to 1 group. Another strength of this study was the choice of qualitative methods (interviews and focus groups). This approach allowed us to gather in-depth information about the experiences of people living with PD and health care professionals regarding their perspectives on the challenges of living with PD. This type of data is difficult to capture through quantitative methods alone, such as surveys or questionnaires. Further, the focus group provided a supportive environment for people living with PD to discuss their experiences and concerns with others who share similar experiences. This approach promoted a sense of community among participants.

The main limitation of this study was the use of an online platform for interviews and focus groups due to the COVID-19 pandemic. While this approach allowed for people living with PD from 2 different regions in Sweden to participate, it also created bias by excluding individuals with limited technological knowledge. Additionally, the recruitment through the Parkinson association limited the possibility of including more isolated and lonelier individuals. Another limitation of the study was the challenge of scheduling a focus group workshop with the same people living with PD who were interviewed initially. Different proposed timings made it difficult to find a suitable time for everyone. Some participants had health conditions that made it challenging for them to attend a 2-hour focus group interview. This situation resulted in a smaller sample size for the focus group than originally intended. Future research can focus on conducting a larger study with a more diverse sample of participants. It could consider recruiting individuals who do not have access to technology or who may be more isolated, to better understand their experiences and perspectives.

### Conclusions

This study showed that the barriers experienced by people living with PD due to PD, such as “fatigue,” “psychological conditions,” “social stigma,” and “medication side effects,” affect their social life. Additionally, people living with PD experience difficulties using keyboards and mouse, remembering passwords, and navigating complex applications due to their physical and cognitive limitations. The people living with PD suggested having a simple and easy-to-use digital community based on their interests and looking for recommendations for events nearby to manage their social isolation and loneliness. Designers need to consider these barriers and facilitators while developing ICT solution among people living with PD. Importantly, any ICT solution designed for people living with PD should not encourage internet addiction, which will further contribute to the person’s withdrawal from society.

## Appendix

Multimedia Appendix 1 Interview guide.

## Abbreviations

ICT: information and communication technology
PD: Parkinson disease
RQ: research question

## Data Availability

The data sets generated and analyzed during this study are not publicly available as they include sensitive data but are available from the corresponding author on reasonable request, including the need of Swedish ethical vetting.
