# Supplementary material for: Information and Communication Technology for Managing Social Isolation and Loneliness Among People Living With Parkinson Disease: Qualitative Study of Barriers and Facilitators
Source: J Med Internet Res. 2024 Jan 17;26:e48175. doi: 10.2196/48175 (PMC10831595; doi:10.2196/48175)
Supplement: Multimedia Appendix 1 [file jmir_v26i1e48175_app1.pdf]

Information and Communication Technology for Managing Social Isolation and Loneliness Among People Living With Parkinson Disease: Qualitative Study of Barriers and Facilitators

Interview Guide

For Healthcare Professionals

1. About Parkinson's disease and Quality of life
    a. Overview of Parkinson's disease among young and older adults.
    b. Differences between the two groups (young and old)
    c. Parkinson's disease and Quality of life
    d. Main complaints from patients.

2. Parkinson's disease and social life (social relationship and social activities)
    a. Relation between Parkinson's disease and social life
    b. Complaints about social life from patients
    c. Reasons for social isolation and loneliness
    d. Impacts of social isolation and loneliness

3. An overview of current assessments and treatments
    a. Any assessment in place for reduced social functioning?
    b. Any treatments in place to manage social isolation?

4. Parkinson's disease and Technology/ICT???
    a. Do you know if the patients use any ICT tools for their social interactions or for social activities?
    b. Do you suggest any kind of ICT tools for them to use as part of routine clinical examinations?
    c. What kind of solutions do you think are needed to improve the social functioning of patients?

For People with Parkinson's (PwPs)

1. Background questions
    a. How old are you?
    b. How long has it been since you have been diagnosed?
    c. Do you work?
    d. What does a typical day look like for you?
2. What challenges do you experience living with Parkinson's disease?
3. Does Parkinson's disease affect your social relationships and social participation? (if so why?)
4. What is social isolation and loneliness for you? What does it mean to you?
5. Do you ever experience social isolation and loneliness? If so, please explain.
6. Questions about social isolation and loneliness (adapted from Cornwell and Waite, 2009):
    a. How large, would you say, your social network is?
    b. How often do you socialize with people in your social network?
    c. In what way do you socialize? (Why?)
    d. What are the different types of social networks do you have? (e.g., spouse, children, friends, neighbor etc.)
    e. How often do you attend meetings of an organized group? (Why?)
        i. In what way do you attend meetings? (Why?)
    f. How many friends do you have?

        i. How often do you socialize with friends?
        ii. How important are friends for you?
        iii. What do you do when you meet?
        iv. How do you socialize with friends?
    g. Do you have family? (size, type)
        i. What is family to you?
        i. How important is family for you?
        ii. What do you do when you meet?
        iii. How often do you socialize with family members?
        iv. How do you socialize with family?
    h. Do you volunteer for any organizations?
        i. How often?
        ii. What do you do?
7. How often do you feel you lack companionship? (Why?)
8. How often do you feel left out? (Why?)
9. How often do you feel isolated from others? (Why?)
10. What can be done to counteract loneliness/feelings of isolation?
11. Could technology support you in managing your social life?
    a. Can technology be a support to counteract social isolation and feelings of loneliness?
    b. Do you have any experience from a technology that is used for counteracting the feelings of loneliness and isolation?
        i. If so, how often and for what?
        ii. If not, why is that?
        iii. If so, what are the problems with those?
        iv. If so, which are the strengths of these technologies?

Focus group discussion topics for PwPs

1. Coping mechanisms for managing social isolation and loneliness
2. What is important to improve social relationship, social participation, and sense of belonging?
3. PD-related challenges to use technology solutions and needs for new solutions
